# Supplementary material for: Influenza A (N1-N9) and Influenza B (B/Victoria and B/Yamagata) Neuraminidase Pseudotypes as Tools for Pandemic Preparedness and Improved Influenza Vaccine Design
Source: Vaccines (Basel). 2022 Sep 14;10(9):1520. doi: 10.3390/vaccines10091520 (PMC9571397; doi:10.3390/vaccines10091520)
Supplement: Supplementary file 1 [file vaccines-10-01520-s001.zip › vaccines-1885052-SM.pdf]

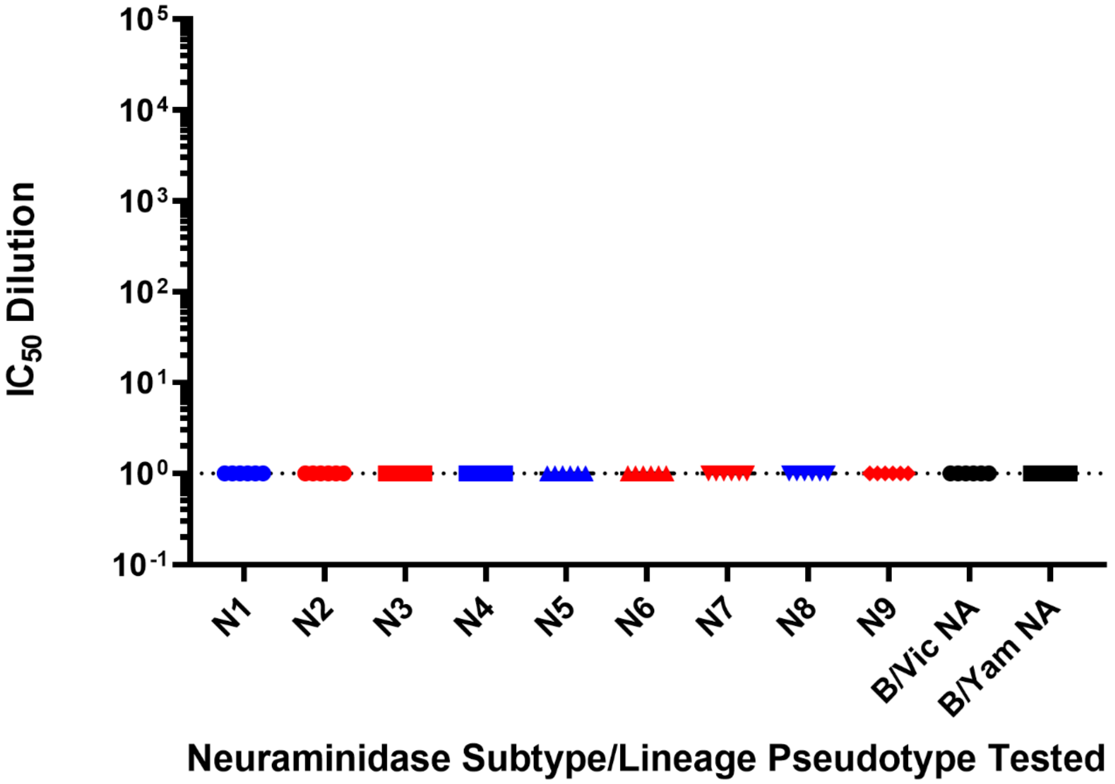

Supplementary Figure S1. Anti-NA activity of mouse sera vaccinated with PBS (negative control) as tested against NA pseudotypes. Sera from mice vaccinated with PBS were tested side by side with those vaccinated with representative IAV and IBV immunogens (Figure 4) against an NA PV panel as indicated: A/Brisbane/2/18 (N1), A/Kansas/14/17 (N2), A/duck/Cambodia/b0116502/17 (N3), A/chicken/NSW/1688/1997 (N4), A/yellow-billed pintail/Chile/C14831/16 (N5), A/yellow-billed teal/Chile/8/13 (N6), A/swine/England/191973/1992 (N7), A/gyrfalcon/Washington/41088-6/14 (N8), and A/Shanghai/2/13 (N9), and Influenza B HA from B/Colorado/6/17 (B/Vic) and B/Phuket/3073/13 (B/Yam). Inhibition was determined via pELLA. Mice that did not show inhibition activity were assigned an arbitrary IC_{50} dilution value of 1 (dashed line) for plotting purposes. Group I NA are indicated in blue and Group II NA in red. (n=6)

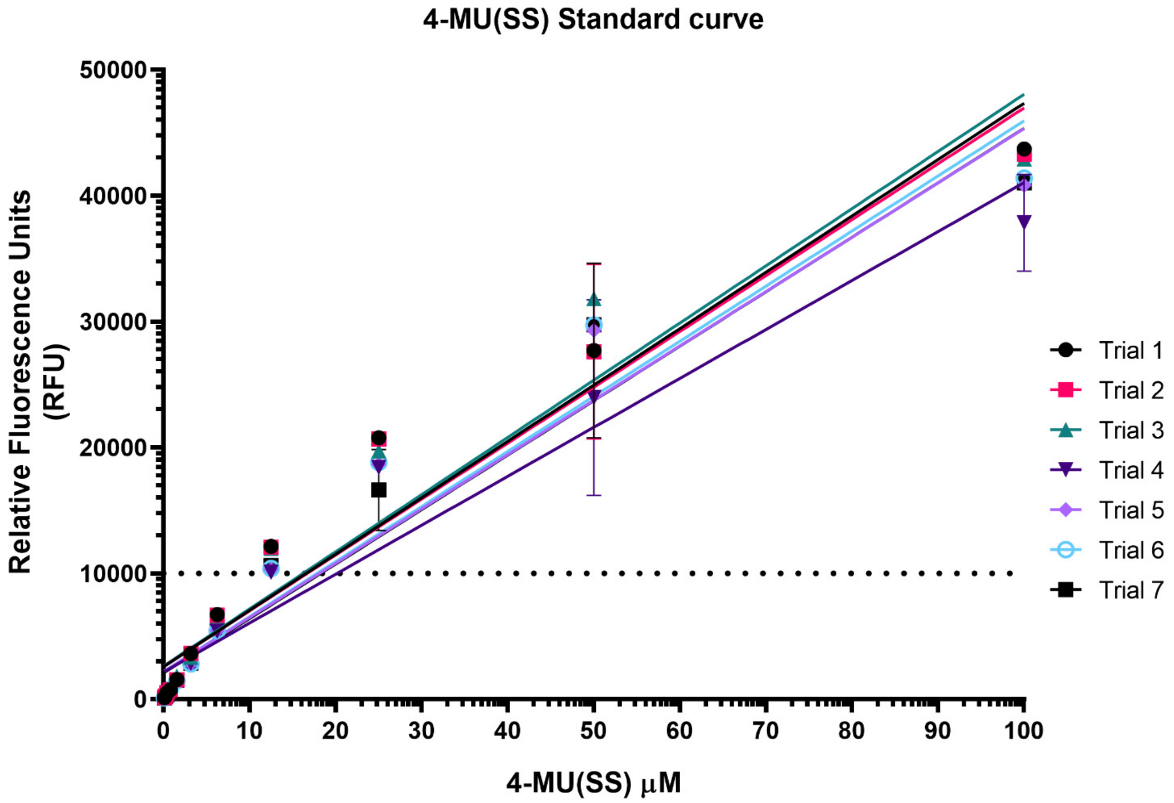

Supplementary Figure S2. Standard Curve of 4-Methylumbelliferone sodium salt (4-MU(SS)). A standard curve was generated with different concentrations of 4-MU(SS) corresponding to a range of RFU values as read via the Tecan Infinite 200Pro fluorescence plate reader at excitation and emission wavelengths of 350 nm and 440 nm respectively (n=7). The Neuraminidase (NA) activity/RFU range for the NA inhibition assay was determined using the linear range of the 4-MU(SS) standard curve. We have arbitrarily chosen ~12 μM 4-MU(SS) corresponding to ~10,000 RFU (broken line) to normalize the NA activities of each PV for use in the NA-Fluor™ NA inhibition assay.

Supplementary Table S1. List of influenza neuraminidase pseudotypes (PV) available at the Viral Pseudotype Unit, University of Kent.

NA Subtype	Strain	Accession #	Plasmid
N1	A/Puerto Rico/8/1934	CY033579	pI.18
	A/California/7/2009	KU933487.1	pI.18
	A/England/195/2009	GQ166659	pEVAC
	A/Brisbane/2/2018	EPI1312565	pEVAC
	A/swine/England/1353/2009	EPI640886	pEVAC
	A/swine/NorthCarolina/A02478985/2020	MT020063	pEVAC
N2	A/Udorn/307/1972	M879361.1	pI.18
	A/Japan/WRAIR1059P/2008(N2)	EPI275488	pEVAC
	A/Korea/KUMC-GR570/2011(N2)	MF441137	pEVAC
	A/Texas/50/2012	KC892281.1	pI.18
	A/chicken/Vietnam/HU3-373/2015	LC426788	pEVAC
	A/Kansas/14/2017	EPI1146344	pEVAC
	A/Switzerland/8060/2017 (N2)	EPI1326014	pEVAC
	A/SouthAustralia/34/2019 (N2)	EPI1607116	pEVAC
	A/chicken/Laos/DC4365/2020	EPI1851379	pEVAC
N3	A/tern/Astrakhan/775/83	AY207523	pEVAC
	A/kelp gull/Chile/C10791/2016 (N3)	MH134604	pEVAC
	A/duck/Cambodia/b0116502/2017	MG591686	pEVAC
N4	A/chicken/NSW/1688/1997	GU053088	pEVAC
	A/red-gartered coot/Chile/C16030/2016	MH675631	pEVAC
N5	A/black duck/AUS/4045/1980	CY005693	pEVAC
	A/yellow-billed pintail/Chile/C14831/2016	MH134707	pEVAC
N6	A/yellow-billed teal/Chile/8/2013	KX101151	pEVAC
	A/duck/Vietnam/HU8-1088/2017(N6)	LC427549	pEVAC
	A/Anhui/2021-00011/2020	EPI1848297	pEVAC
	A/duck/NgheAn/5382VTC/2019	EPI1665322	pEVAC

N7	A/duck/Manitoba/1953	KF435051	pEVAC
	A/swine/England/191973/1992	U85988	pEVAC
N8	A/gyrfalcon/Washington/41088-6/2014 (N8)	EPI569392	pEVAC
N9	A/Shanghai/2/2013	EPI448938	pEVAC
	A/Gansu/23275/2019	EPI1431607	pEVAC
N10	A/Little shouldered bat/Guatemala/060/2011	CY103894.1	pI.18
N11	A/flat-faced bat/Peru/033/2010	CY125947	pEVAC
B/Vic NA	B/Colorado/6/2017	EPI969379	pEVAC
	B/Washington/2/2019	EPI1368872	pEVAC
B/Yam NA	B/Phuket/3073/2013	EPI1349898	pEVAC
